# Recent advances in MASLD genetics: Insights into disease mechanisms and the next frontiers in clinical application

**DOI:** 10.1097/HC9.0000000000000618

**Published:** 2025-01-07

**Authors:** Vincent L. Chen, Graham F. Brady

**Affiliations:** Division of Gastroenterology and Hepatology, Department of Internal Medicine, University of Michigan, Ann Arbor, Michigan, USA

**Keywords:** genetic risk, GWAS, outcome prediction, pathophysiology, steatotic liver disease

## Abstract

Metabolic dysfunction–associated steatotic liver disease (MASLD) is the most common chronic liver disease in the world and a growing cause of liver-related morbidity and mortality. Yet, at the same time, our understanding of the pathophysiology and genetic underpinnings of this increasingly common yet heterogeneous disease has increased dramatically over the last 2 decades, with the potential to lead to meaningful clinical interventions for patients. We have now seen the first pharmacologic therapy approved for the treatment of MASLD, and multiple other potential treatments are currently under investigation—including gene-targeted RNA therapies that directly extend from advances in MASLD genetics. Here we review recent advances in MASLD genetics, some of the key pathophysiologic insights that human genetics has provided, and the ways in which human genetics may inform our clinical practice in the field of MASLD in the near future.

## INTRODUCTION

Over the last 15–20 years, our understanding of the underlying genetics and pathophysiology of metabolic dysfunction–associated steatotic liver disease (MASLD) has exploded, largely due to a convergence of simultaneous advances in human genetics, including genome-wide association studies (GWAS), and in vivo techniques for modeling human disease in animal models over the same period. In this review, we highlight key studies in the field and discuss how human genetics have contributed to our understanding of MASLD pathophysiology. In addition, we explore how advances in MASLD genetics are starting to enter clinical practice and how they may inform clinical hepatology, with an eye toward personalized medicine, in the years to come.

## ADVANCES IN MASLD GENETICS

The first common MASLD-promoting variant to be identified through GWAS for hepatic steatosis by magnetic resonance spectroscopy was *PNPLA3*-rs738409-G, corresponding to the I148M coding variant.^1^ Subsequent studies based on imaging and/or histology identified variants in/near *TM6SF2*, *MBOAT7*, *GCKR*, *LYPLAL1*, and *PPP1R3B*,^2^^–^^5^ though the last was ultimately found to be primarily a glycogen storage–modulating variant.^6^ More recently, a splice variant in *HSD17B13* that results in a defective protein was found to be *protective* against diverse etiologies of chronic liver disease,^7^ and a distinct variant in high linkage disequilibrium with the splice variant (ie, likely the same genetic signal) also associated with histologic improvements in metabolic dysfunction–associated steatohepatitis (MASH).^8^

Several more recent studies published within the last 5 years have further expanded upon these findings (Table 1). Among these, 1 study from the nationwide multiethnic Million Veterans Program evaluated a surrogate measure of hepatic steatosis defined by chronic ALT elevation,^67^ while another study meta-analyzed imaging-based cohort studies (Genetics of Obesity-related Liver Disease consortium and UK Biobank MRI-PDFF) with diagnosis code-based steatosis cohorts (UK Biobank, FinnGen, and electronic MEdical Record and GEnomics [eMERGE]). Together, these 2 studies identified several new variants in genes involved in lipid and glucose biology including *TRIB1*, *MARC1*, *MTTP*, *PPARG*, *APOE*, and *APOH*. Both studies also identified associations of ﻿these variants, and polygenic risk scores summing their combined effects, with ﻿hepatic steatosis, diverse cardiometabolic traits, and more advanced liver disease, including cirrhosis and HCC.

**TABLE 1:** Genetic variants associated with steatotic liver disease

Gene	Associated traits	Putative disease mechanisms
*PNPLA3*	Steatosis^1^,^,^^9^^–^^13^ Fibrosis/cirrhosis^14^^–^^19^ Decompensation^16^^,^^20^^,^^21^ HCC^22^^–^^24^	Impaired triglyceride export from the liver. HSC activation.^25^ Mutated PNPLA3 protein may interact with PNPLA2 protein (see *PNPLA2* entry below).^26^
*TM6SF2*	Steatosis^9^^,^^10^^,^^27^ Fibrosis/cirrhosis^14^,^,^^15^^,^^19^^,^^28^^–^^30^ Decompensation^31^^,^^32^ HCC^24^^,^^30^^,^^33^	Impaired triglyceride lipidation and export from the liver, especially large triglyceride-rich VLDL^34^^–^^36^
*HSD17B13*	Steatosis^7^^,^^8^ Steatohepatitis^7^^,^^8^^,^^37^ Fibrosis/cirrhosis^8^^,^^15^^,^^19^^,^^30^ HCC^30^	Localizes to lipid droplets and has retinol dehydrogenase activity.^7^^,^^8^ Knockout increased expression of fatty acid synthesis–related proteins and impaired beta-oxidation.^38^
*MARC1*	Steatosis^9^^,^^10^^,^^39^ Steatohepatitis^40^ Cirrhosis^28^,^,^^39^^–^^41^	Likely due to changes in hepatocyte secretion of VLDLs. mARC1 knockdown in vitro in human hepatocytes reduces lipid accumulation; hepatocyte-specific knockdown in vivo in mice reduces steatosis/steatohepatitis^42^
*TOR1B*	Steatosis^9^^,^^43^ Fibrosis/cirrhosis^19^^,^^28^	Unclear. Torsin knockouts in mice lead to decreased lipid secretion from cells, increased intracellular lipids, and decreased circulating limits.^44^
*MBOAT7*	Steatosis^9^^,^^10^^,^^45^ Fibrosis/cirrhosis^28^^,^^45^ HCC^46^	Increased de novo lipogenesis and inflammation through SREBP-1c activation and fatty acid content of phosphoinositols.^47^
*APOE*	Steatosis^10^^,^^48^^,^^49^ Cirrhosis^19^^,^^28^	APOE protein increases HDL uptake into the liver and metabolizes retinols.^50^
*ADH1B*	Steatosis^10^^,^^51^^,^^52^ Cirrhosis^28^	Codes for an alcohol dehydrogenase, the minor allele of ﻿which results in impaired alcohol metabolism. May have distinct effects depending on the degree of alcohol consumption: protective in non-drinkers and harmful in drinkers.^51^^,^^52^
*GPAM/GPAT1*	Steatosis^9^^,^^10^^,^^49^ Cirrhosis^28^	Catalyzes the initial step of triglyceride synthesis. *Gpam* overexpression or knockout in mice leads to increased or decreased liver fat content, respectively.^53^^,^^54^ Mechanisms underlying associations between genetic variants are not well-characterized
*TRIB1*	Steatosis^10^^,^^55^ Cirrhosis^28^	Likely glucose-dependent de novo lipogenesis. Mice without *Trib1* have increased fatty acid synthesis.^56^
*GCKR*	Steatosis^9^^,^^10^^,^^13^^,^^57^	GCKR is a negative regulator of glucokinase. Defective GCKR protein results in increased glycolysis and downstream processes including de novo lipogenesis.^58^^,^^59^
*MTTP*	Steatosis^9^^,^^10^^,^^60^^,^^61^ Cirrhosis^28^	Normal gene increases transport of phospholipids and triacylglycerols to ApoB for assembly of lipoproteins that can be exported from the liver. Liver-specific mutations reduce serum atherogenic lipid concentrations but cause hepatic steatosis.^62^^–^^64^ Deficiency causes abetalipoproteinemia.^65^
*PNPLA2/ATGL*	Steatosis^9^^,^^10^	PNPLA2/ATGL promotes lipolysis through ABHD5. PNPLA3 competes with PNPLA2/ATGL for binding to ABHD5 and mutated PNPLA3 is more effective at this competition, resulting in decreased ABHD5-dependent lipolysis^26^
*INSR*	Steatosis^10^^,^^66^	MASLD-promoting variants in *INSR* are associated with increased hepatic insulin resistance.^66^

There is increasing recognition of the value of GWAS not only for steatosis but also for more advanced liver disease, particularly steatohepatitis and fibrosis/cirrhosis. Most GWAS for cirrhosis/fibrosis have either focused on alcohol-associated liver disease^14^^,^^28^^,^^41^^,^^68^^,^^69^ or included any etiology of cirrhosis.^15^^,^^39^ However, some histologic and imaging studies have assessed for more advanced disease specifically in patients with MASLD. A recent GWAS of histologic MASLD was conducted in the United Kingdom, Switzerland, Belgium, France, Germany, and Italy, including 1483 mostly middle-aged participants with a high prevalence of type 2 diabetes (40%) and obesity (median body mass index, 35 kg/m^2^); 10% had cirrhosis, and 56% had steatohepatitis. Consistent with previous studies, variants in *PNPLA3*, *TM6SF2*, *GCKR*, and *HSD17B13* were associated with MASLD.^70^ When evaluating variants associated with steatohepatitis, the *PNPLA3* variant and a novel variant in *LEPR* were associated with steatohepatitis, with suggestive signals in *GCKR*, *TM6SF2*, and *HSD17B13* that did not reach genome-wide significance.^70^ Similarly, *PNPLA3*, *TM6SF2*, and *GCKR* variants are significantly associated with advanced fibrosis.^70^ However, histologic studies have been limited by a relatively small sample size and few cases of cirrhosis, resulting in limited power.

Given the limitations of histologic studies, there has been interest in noninvasive surrogates of steatohepatitis and/or fibrosis. One recent GWAS assessed for variants associated with corrected T1 time, a measure of extracellular hepatic water content that correlates with fibrosis and inflammation, in 14,440 participants.^71^ As expected, variants in *PNPLA3* and *TM6SF2* were associated with corrected T1 time, but this study found novel variants in *SLC39A8* and *SLC30A10* that were associated not only with corrected T1 time but also with aminotransferase concentrations, implying a potential role in steatohepatitis. Of note, *SLC39A8* encodes ZIP8, which is a divalent cation importer that may regulate manganese, zinc, and selenium uptake, which are all important in hepatic inflammation, and *SLC30A10* also encodes a gene involved in manganese transport. Thus, the identification of these variants could imply a link between divalent cations and steatohepatitis. However, caution is warranted in the interpretation of these findings, and the relationship between divalent cations and MASLD is unclear; for example, while manganese supplementation could be protective against MASLD,^72^ excess manganese is hepatotoxic.

Beyond GWAS, the most recent exciting advance in genetics is whole-exome sequencing and gene-based analyses. Classic GWAS are variant-based analysis, in which one determines whether *individual* genetic *variants* are associated with a trait. In gene-based analyses, one sums the effects of deleterious coding variants in a gene and determines the *collective* (not individual) burden of these variants to determine the effect of a *gene* on a trait. This is relevant when individual disease-causing variants are too rare to allow for adequate power to detect associations^73^ and usually requires whole-exome sequencing since genotyping arrays often cannot accurately identify rare variants. One recent example of this identified genes associated with both AST and ALT based on whole-exome sequencing in a cohort of >540,000 primarily community-based participants from the United Kingdom, Sweden, and the United States. They then validated several of these variants on an endpoint of chronic liver disease.^74^ They found that rare coding variants in the *CIDEB* gene were protective against diverse etiologies of chronic liver disease in independent cohorts and were also associated with improved liver histology in patients undergoing liver biopsy.^74^ Subsequent cell line work showed﻿ that Huh7 cells treated with oleic acid (to induce lipid droplet formation) tended to generate smaller droplets if they were also treated with a small inhibitory RNA targeted against *CIDEB* than if treated with a control small interfering RNA.^74^

GWAS has been a powerful tool for understanding the genetic basis for human disease but is only a first step, and further downstream analyses must be conducted to translate in silico genomic findings into biologically meaningful results. In our opinion, the advances from GWAS that are most likely to impact care in the next 15–20 years are as follows: first, insight into pathophysiology and disease biology derived from GWAS hits; second, clinical risk stratification in patients both with cirrhosis (especially HCC risk) and without cirrhosis (including the potential to add value beyond fibrosis stage and noninvasive scores such as Fibrosis-4 [FIB4]); and third, precision health in which one’s genotype might contribute to decisions surrounding therapy.

## INSIGHTS INTO DISEASE PATHOPHYSIOLOGY (BEDSIDE TO BENCH—AND BACK)

**FIGURE 1:**
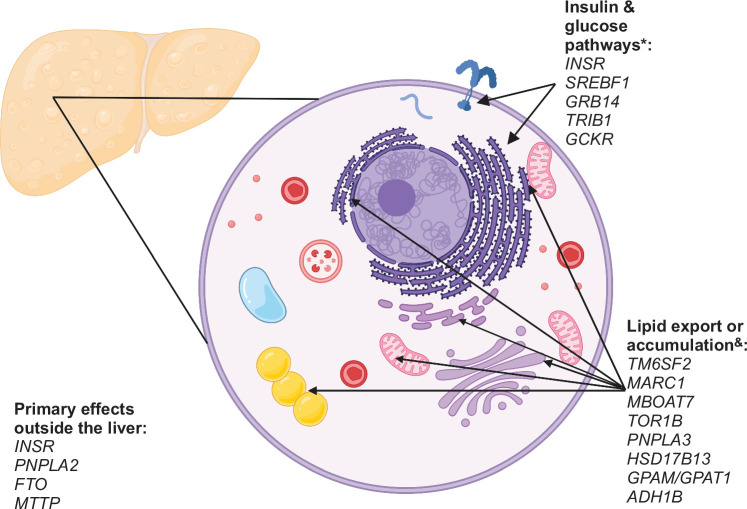
Metabolic pathways impacted by MASLD-linked genes. The major hepatocyte metabolic pathways that are thought to be affected by MASLD-linked genes and the primary cellular organelles corresponding to those pathways are shown. The few MASLD-linked genes that are thought to primarily impact MASLD susceptibility through extrahepatic effects are listed separately in the bottom-left corner. *Variants in these genes are also strongly associated with cirrhosis; ^&^variants in these genes are not strongly associated with cirrhosis but impact cardiovascular risk. Created in BioRender. Abbreviation: MASLD, metabolic dysfunction–associated steatotic liver disease.

While other types of studies, including mouse models and human liver transcriptome studies, have contributed in important ways to our understanding of MASLD pathophysiology, human genetics—and in particular GWAS—have dramatically enhanced our understanding of MASLD over the last 15–20 years. The strongest common MASLD-promoting variant identified to date, *PNPLA3*-rs738409-G corresponding to I148M, was initially thought to potentially be a loss-of-function variant; however, landmark knockout mouse studies established that this was not the case, as the *Pnpla3*-knockout mouse did not develop MASLD/MASH.^75^ Later studies of knock-in *Pnpla3* I148M mice, coupled with in vitro studies of wild-type and I148M PNPLA3 biology, have demonstrated the role of PNPLA3 as a lipid droplet–binding protein and the deleterious metabolic consequences of failed lipid droplet catabolism due to nondegradable PNPLA3 I148M.^76^^–^^78^ In addition to revealing the importance of lipid droplet turnover in MASLD, the linkage of *PNPLA3*-rs738409-G to both MASLD and alcohol-associated liver disease has shed light on the common pathways of liver injury seen in these 2 distinct types of chronic liver disease. The convergent pathophysiology through lipid metabolism in MASLD and alcohol-associated liver disease has been further reinforced by the linkage of *MBOAT7-*rs641738 to both diseases, given that *MBOAT7* encodes a membrane-bound lipid acyltransferase.^5^^,^^14^ Further demonstrating the importance of hepatocyte lipid metabolism, catabolism, and export in protection from MASLD, the common variant with the second-strongest effect size is *TM6SF2* E167K (rs58542926). This variant results in loss of function and impairs VLDL export, resulting in increased hepatic lipid content and decreased circulating lipids; this finding in human studies has been confirmed in vitro and in animal models, including in zebrafish.^14^^,^^79^^,^^80^ Similar findings, further enforcing the importance of lipid export in protection from MASLD, have been seen with *APOE* variants that predispose to liver disease but may be protective against Alzheimer’s disease^48^ and an *MLXIPL* variant that associates with reduced serum triglycerides but has a higher risk of MASLD.^81^ Lastly, and perhaps most importantly, these human genetics studies establishing the roles of these lipid metabolism and export genes in MASLD have also firmly established the role of the hepatocyte in MASLD pathogenesis (Figure 1). While MASLD is epidemiologically linked to extrahepatic traits including type 2 diabetes, obesity, and insulin resistance, these genetic studies have provided evidence that the hepatocyte is not simply a bystander in hepatic steatosis and MASLD; this fundamental insight provided by human genetics has subsequently been confirmed directly by a large number of animal studies utilizing hepatocyte-specific knockout or knock-in techniques.

Expanding upon the role of hepatocyte lipid handling in MASLD, 2 recent studies have proposed dividing variants based on directions of effect on decreasing hepatic lipid export versus other mechanisms such as increased de novo lipogenesis.^9^^,^^82^ This study found that the first group—dominated by *PNPLA3* and *TM6SF2* variants—is associated with lower serum triglycerides and either protection or no association with cardiovascular disease but markedly increased risk of advanced liver disease, including HCC. In contrast, the second group is associated with higher serum triglycerides and a much greater risk of cardiovascular disease, with a more modest association with advanced liver disease. The 2023 Chen et al^10^ study characterized the 17 variants they identified into even more granular categories based on disease biology and effect on cardiometabolic traits, including a low lipoprotein output category (comprising variants in *PNPLA3*, *TM6SF2*, and *PTPRD*) whose disease biology is primarily related to decreased hepatic export of lipids from the liver. Another category comprises variants in *TOR1B*, *ADH1B*, *MBOAT7*, *GPAM*, and *MARC1* that increase intrahepatic lipid load by increasing storage and/or production of intrahepatic lipids, while not directly influencing lipid export. Other categories consisting of *GCKR*, *TRIB1*, *GRB14*, and *SREBF1* variants increase hepatic de novo lipogenesis; *TOR1B* and *ADH1B* may promote storage rather than production of de novo lipogenesis products. Still other variants likely exert their effects largely outside of the liver, such as variants in *INSR* and *PNPLA2* that increase the release of fatty acids from adipose tissue; lipid absorption in and transport from the intestine (*MTTP*); and abnormal adipocyte activation (*IRX3/5* and *FTO*, which is the strongest obesity-promoting variant and likely influences hepatic steatosis indirectly.^83^)

Identification of genetic drivers of the epidemiologically well-recognized pattern of insulin resistance, type 2 diabetes, and MASLD has been somewhat more complicated than linkages to MASLD itself. For example, while variants in genes such as *PNPLA3* and *TM6SF2* strongly predispose to MASLD, they may even protect against hyperlipidemia and cardiovascular disease, as noted above. Thus, complementary metabolic traits–focused GWAS and familial studies have helped to fill in these important gaps. For example, the linkage of *GCKR* to multiple metabolic traits and *LYPLAL1* to central obesity, along with MASLD, has helped to firmly establish the causal link between metabolic dysfunction, adipose tissue distribution, and MASLD. Toward that end, studies of families with severe and early-onset insulin resistance, diabetes, and MASLD associated with aberrant adipose tissue distribution (lipodystrophy syndromes) have broadened our understanding of metabolic dysfunction and MASLD pathogenesis over the past 20 years. Among these have been linkages of *PPARG*, *CIDEC*, and *LMNA*, among other genes, to congenital partial lipodystrophy syndromes.^84^^–^^87^ The last of these is particularly interesting because it encodes a structural nuclear protein (lamin A/C) that is ubiquitously expressed in all tissues and does not have an obvious functional linkage to lipid metabolism. However, in vitro and mouse studies have directly or indirectly confirmed the pathogenic impact of *LMNA* variants in both adipocytes and hepatocytes in driving lipodystrophy and MASLD, thus establishing that the nuclear envelope and lamina play important roles in protecting against metabolic disease.^88^^–^^90^ While the precise mechanisms by which *LMNA* variants predispose to metabolic disease in humans remain incompletely understood, more recent human genetics and mouse studies have linked additional structural nuclear proteins including LAP1, LAP2, TOR1B, and SUN1 to hepatic steatosis and MASLD as well.^10^^,^^44^^,^^91^^,^^92^ Thus, these small studies of families with monogenic severe metabolic disease have opened up an entire area of investigation regarding the roles of structural nuclear proteins in protection from metabolic disease.

The last area in which human genetics has opened up new areas of investigation and sheds important light on aspects of MASLD biology is the area of fibrosis and progression to cirrhosis. It is well-recognized that fibrosis progression is the primary and most important driver of morbidity and mortality in chronic liver disease, but there is significant variation among MASLD-associated genetic variants in terms of fibrosis risk. Of particular interest in this regard is *PNPLA3*, which associates strongly with fibrosis in addition to steatosis.^93^^,^^94^ This observation led to the finding that this gene is also expressed in HSCs, where it likely plays a direct role in retinol metabolism, stellate cell activation, and hepatic fibrosis.^95^^,^^96^ Not only does this human genetics–informed finding expand our understanding of MASLD progression, but it also has potentially significant implications for the clinical implementation of PNPLA3-targeted therapies in the future, as these will likely need to be delivered to multiple cell types, including stellate cells, rather than only to hepatocytes. In contrast, *HSD17B13* is similarly linked strongly to fibrosis protection, but it is not strongly linked to steatosis, and its expression is highly specific to hepatocytes.^7^ These concurrent findings provided immediate and important insights into MASLD biology and the role of the hepatocyte in propagating liver injury and fibrosis. Precisely how the *HSD17B13* loss-of-function variant protects from hepatic fibrosis progression remains incompletely understood, but this is an active area of investigation, and the encoded protein appears to be a promising therapeutic target to prevent fibrosis in all forms of chronic liver disease regardless of the underlying etiology.

## CLINICAL APPLICATIONS

**FIGURE 2:**
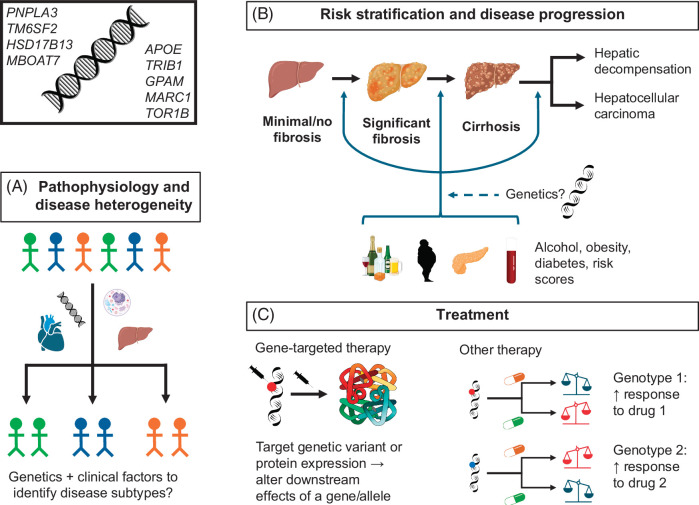
Potential clinical applications of genetics in liver disease. Top left illustrates selected genes in which variants have been associated with MASLD. (A) A heterogeneous population of patients with MASLD with distinct underlying pathophysiology and/or clinical manifestations (depicted by differences in colors) can be subdivided into more uniform disease subtypes based on genetics, liver and cardiac phenotypes, and cellular mechanisms. (B) MASLD can progress from minimal/no fibrosis to significant fibrosis to cirrhosis and, ultimately, decompensation and HCC. The primary known predictors of progression are alcohol intake, cardiometabolic comorbidities, including obesity and diabetes, and risk scores such as Fibrosis-4; genetics may also predict the risk of progression between these disease stages. (C) Treatment for MASLD can be broadly divided into gene-targeted therapy, which is directed at a genetic variant or expression thereof, to affect downstream effects of a gene/allele, or other therapy, which is not directly targeted at specific genotypes. In the case of other therapies, certain genotypes may hypothetically still be associated with differential response to treatment. Note, however, that none of these potential applications of human genetics is yet ready for use in clinical practice or endorsed by professional societies Elements of this figure were created in BioRender. Abbreviation: MASLD, metabolic dysfunction–associated steatotic liver disease.

While there are several potential opportunities for the incorporation of genetics into clinical practices, we will focus on (1) risk stratification in patients with or without cirrhosis, and (2) treatment, either with “gene therapy” directed at specific genetic variants or by identifying which patients are most likely to respond to treatment (Figure 2).

### Risk stratification: Patients with noncirrhotic MASLD

Risk stratification within noncirrhotic MASLD is perhaps the greatest opportunity for clinical application of human genetics, given that this group represents the vast majority of patients with MASLD, existing prediction tools are imperfect, and most patients will progress slowly or not at all.^97^^,^^98^ The *PNPLA3* risk allele is associated with the incidence of major adverse liver-related outcomes (MALO) in persons with MASLD or cardiometabolic risk factors.^20^^,^^21^^,^^99^^,^^100^ Importantly, this risk is independent of the histologic fibrosis stage. In 2075 MASH Clinical Research Network participants, PNPLA3-rs738409-G was associated with an increased risk of MALO, with a subhazard ratio of 1.51 and 1.94 for CG and GG genotypes versus CC, respectively. This difference was greater in individuals with advanced fibrosis (cumulative incidence 85% vs. 58% in those with vs. without the G allele) than those without it (corresponding cumulative incidence 25% vs. 8%).^20^ Similarly, a multicenter Japanese cohort of 1178 patients also found an association between *PNPLA3* genotype and MALO in the overall cohort, again with a much greater delta in cumulative 10-year MALO events in patients with advanced fibrosis (>50%) than in those without advanced fibrosis (delta <20%).^31^

Biopsy is becoming much less frequently employed in MASLD, and it will be crucial to determine whether the *PNPLA3* genotype adds significantly to other types of noninvasive risk stratification. The most widely recommended clinical risk scores include AST-platelet ratio index and FIB4, which are endorsed by AASLD, EASL, and AGA to stratify risk for MALO in MASLD,^101^^–^^103^ and the rate of change in FIB4 is a prognostically important noninvasive biomarker that is in part genetically-influenced.^104^ Whether genetics add to the AST-platelet ratio index and FIB4 may depend on the context. Innes et al^105^ systematically reviewed blood-derived scores for liver fibrosis and calculated their accuracy for predicting the 10-year risk of MALO in UK Biobank (a community-based cohort), with or without including a genetic risk score. They found little incremental benefit of incorporating genetics into high-performing scores such as FIB4 (C-statistic 0.78–0.79 without vs. with genetics) and AST-platelet ratio index (C-statistic 0.80–0.81). However, other studies have suggested the potential benefit of genetics. Another study used an Italian cohort of 546 patients with either histologic MASLD or vibration-controlled transient elastography >11–11.5 kPa and at least 1 metabolic syndrome criterion and developed a combined genetic and metabolic staging system, including *PNPLA3*, *TM6SF2*, and *HSD17B13* genotypes along with age, sex, thrombocytopenia, diabetes, hypoalbuminemia, and low HDL concentration.^106^ This yielded 5 risk classes with a 5-year cumulative incidence of MALO ranging from 4% (eg, a woman under 55 years old without any of the clinical or genetic risk factors) to 90% (eg, a man over 65 years old with platelet count <110,000/mm^3^, type 2 diabetes, serum albumin <4 mg/dL, and all genetic risk variants).^106^ They externally validated this score in UK Biobank participants with FIB4 ≥1.3 and found a >20-fold range of risk of severe liver disease.^106^ Another study^16^ added *PNPLA3* genotype to diabetes status (a major predictor of clinical outcomes in MASLD) in individuals with suspected MASLD based on chronic ALT elevations from both a US hospital–derived cohort and the community-based UK Biobank.^107^^,^^108^ In the intermediate-risk FIB4 category (1.3–2.67), participants with both highest risk *PNPLA3* genotypes (rs738409-GG) and diabetes had an overall incidence of cirrhosis that was not significantly different from that of individuals with high FIB4 (>2.67). In other words, the presence of diabetes and at-risk *PNPLA3* genotype elevated one’s risk category beyond what was predicted by FIB4 alone.

Thus, genetics, including the *PNPLA3* genotype, appears to improve risk stratification beyond blood-based scores and histology. One notable gap in the literature is that there are minimal data on incremental benefit of genetics to imaging-based elastography.^109^ As vibration-controlled transient elastography and magnetic resonance elastography are more widely used in clinical practice, future studies will need to address this limitation.^102^^,^^110^ There are also limited data on the patient's perspective on genetic risk, with one study showing a wide range of responses, from feeling less at fault for having MASLD to experiencing increased anxiety about prognosis.^111^

### Risk stratification: Patients with cirrhosis

*PNPLA3* genotype may also help predict HCC in the key populations of cirrhosis and at-risk chronic hepatitis B; however, there are minimal data on genetics and longitudinal outcomes in chronic hepatitis B^112^^,^^113^ so we will focus on cirrhosis for the rest of this discussion. We note that the highest-quality studies are not in MASLD-related cirrhosis specifically.

Several studies have assessed the impact of genetics on HCC risk prospectively. One French prospective study assessed *PNPLA3* genotype in >500 patients with HCV or alcohol-associated cirrhosis.^22^ In the alcohol-associated cirrhosis cohort, the *PNPLA3* risk allele was associated with HCC, but there was no significant association in the HCV cohort.^22^ A more recent study also assessed the *PNPLA3* genotype in 2 prospective French and Belgian cohorts, one with cured HCV cirrhosis^114^ and another with alcohol-associated cirrhosis,^115^ with a total of 1145 patients.^116^ They generated polygenic risk scores, including variants in *PNPLA3*, *TM6SF2*, *MBOAT7*, *HSD17B13*, and *APOE* that were associated with the incidence of HCC, even after adjustment for age-male--albumin-bilirubin-platelets score and an internally derived risk score for HCC.^116^^,^^117^ However, there was a modest benefit of incorporating genetics, with a C-statistic of 0.77 for the clinical models alone versus 0.78–0.79 for clinical and genetic models. Another recent prospective US study of 1911 patients with cirrhosis found that *PNPLA3*-rs738409-G carriers had a higher risk of HCC, with a greater effect in those with heavy alcohol intake, obesity, or viral hepatitis.^23^ Combining the *PNPLA3* genotype with a recently developed model incorporating several parameters, including AFP,^118^^,^^119^ resulted in a marked increase in C-statistic for 1-year HCC prediction, which﻿ increased from 0.78 to 0.83. A number of retrospective studies have also demonstrated associations between HCC and genetic variants including *PNPLA3*, *TM6SF2*, and *HSD17B13*,^24^,^,^^120^^–^^122^ mostly in cohorts with cirrhosis from any cause, though some of the studies conducted subgroup analyses in MASLD-related cirrhosis.

### Precision therapy

We will briefly discuss how genetics may inform pharmacologic therapy. One approach is to develop treatments directed at correcting the pathophysiologic process caused by a specific variant or group of variants. This approach may take several forms, including small inhibitory RNA or adenovirus vectors, and has proven highly effective in conditions including sickle cell disease, spinal muscular atrophy, and hemophilia.^123^^–^^125^ There are at least 2 ongoing phase II clinical trials in MASH evaluating gene therapy. A randomized placebo-controlled trial is investigating the impact of an antisense oligonucleotide in patients with MASH and homozygosity for the *PNPLA3* variant (NCT05809934). Another study compares a small interfering RNA agent designed to knock down *HSD17B13* expression (based on the association of loss-of-function variants in *HSD17B13* with protection from chronic liver disease) to a placebo (NCT05519475). Both studies are ongoing, and given the complexity of MASLD/MASH pathogenesis in comparison to Mendelian disorders such as sickle cell disease, it remains to be seen how effective gene therapy for MASH might be.

Genetic variants could also impact responses to existing therapies that are not targeted against genetic variants, either because certain genetic variants globally increase disease risk (and thus allow for treatment effects to become more apparent), or because they could impact disease pathophysiology. For example, if persons carrying a certain allele or group of alleles are more likely to have disease pathophysiology driven by liver-specific lipid accumulation (eg, the pathophysiologic process characteristic of *PNPLA3* and *TM6SF2* risk variants) versus systemic glucose or insulin metabolism (eg, the pathophysiologic process more characteristic of *GCKR, TRIB1*, and *SREBF1* variants), they could potentially be more likely to respond to liver-specific mechanisms (such as thyroid receptor-β agonists) or systemic treatments such as glucagon-like peptide 1 receptor agonists, respectively. There are limited data on the impact of genetics on response to treatment. In a systematic review, carriage of the *PNPLA3* risk variant was inconsistently associated with improved response to lifestyle interventions, but with decreased response to omega-3 fatty acids and dapagliflozin.^126^ Most of these studies were small randomized or observational trials using magnetic resonance spectroscopy or proton density fat fraction as the outcome.^126^ Another study found that among 220 individuals who were started on semaglutide (for approved indications of diabetes and obesity, not for MASLD), those ﻿carrying at least 1 ﻿*PNPLA3* risk allele experienced larger decreases in ALT afterward and were more likely to develop a clinically relevant decrease in ALT of ﻿≥17 U/L, which has been previously shown to correlate with histologic response.^127^^,^^128^ This study was limited by reliance on the surrogate endpoint of ALT. All studies on this topic have been small, and these findings will have to be validated prospectively, ideally in the context of randomized controlled trials.

## WHAT IS ON THE HORIZON FOR MASLD GENETICS AND GENETICS-INFORMED DISEASE MANAGEMENT?

Advances in our understanding of MASLD pathophysiology have exploded in the last 15–20 years, largely due to human genetics–informed hypothesis-driven basic and translational research. While it is unlikely that additional MASLD-promoting common variants that have large effects like those in *PNPLA3* and *TM6SF2* will be revealed by future genetic studies, it is very likely that additional variants that have smaller but still important effects (either protective or deleterious), or rare variants or groups of variants that have a strong effect, will continue to be uncovered as larger and larger cohorts, with deeper phenotyping and higher-resolution genomic data, come online. Indeed, we have already seen novel and important variants revealed by the incorporation of large data sets with liver biopsy or MRI data within the last few years.^10^ In our view, though, the future of MASLD genetics and genetics-informed clinical practice can largely be broken down into 2 major categories: (1) functional and mechanistic characterization and (2) clinical application.

Further mechanistic delineation of how MASLD-linked genes and variants functionally drive MASLD pathogenesis and/or progression will be crucial. Such basic and fundamental studies are absolutely critical in order for new gene-, variant- and pathway-targeted therapies to be successfully developed and implemented. For example, it was from in vitro and mouse studies that we learned that *PNPLA3*-rs738409-G is a gain-of-function variant and that targeting *PNPLA3* expression through RNA interference may be a fruitful strategy; without such mechanistic insights from the laboratory, translating human genetics into novel therapies is not possible. Thus, basic studies in animals including mice, zebrafish, and other models, as well as mechanistic studies in humans, will be critical steps in translating many of our genetic discoveries from the last 20 years into new MASLD therapies in the next 20–30 years.

We envision clinical applications of MASLD genetics being divided into 2 broad categories—one of which is nearly ready for implementation: (1) prognostication and prediction of therapeutic response; (2) gene/variant-targeted therapy versus nontargeted therapy (with potential for combination therapy). Our group and others have recently demonstrated as proof-of-concept that currently available genetic data, particularly the *PNPLA3* genotype, can be used to predict both patient outcomes and the likelihood of response to treatment. Barriers to the implementation of genetic testing in the clinic currently include (1) uncertainty regarding the willingness of insurance carriers to reimburse such testing and of patients to undergo genetic testing (eg, if it may result in discrimination by insurance carriers) and (2) guidance on how genetic testing results would affect management. The first barrier will require advocacy and policy work. We envision the second barrier being addressed through new drugs— for example, if targeted therapies such as *PNPLA3* and/or *HSD17B13* RNA interference are eventually approved for MASLD. In addition, if genetics can be consistently shown to have significant prognostic value, it may help expand clinical indications for drug therapy: for example, if subgroups of patients with stage 1 fibrosis have a similar 10-year risk of MALO as the average patient with stage 2 fibrosis, but this risk can be mitigated with a drug, these patients could be included in clinical trials and potentially derive meaningful benefit from pharmacologic therapy. If, as expected, additional pharmacologic agents become available for MASLD within the next 10 years, identification of which patients are most likely to benefit from a medication, or a combination of medications, will become increasingly valuable; human genetics has great potential in this area, and thus we envision it becoming part of how personalized hepatology care is delivered in the 21st century.
